# Observational Study on Enhancing End-of-Life Care Competence Through OSCE: Educational Implications

**DOI:** 10.1007/s40670-024-02275-7

**Published:** 2025-01-09

**Authors:** Juan Mora-Delgado, Cristina Lojo-Cruz, Manuel J. Bández, Manuel Rosety-Rodríguez, Ángel Estella García

**Affiliations:** 1 Internal Medicine and Palliative Care Clinical Management Unit, Hospital Universitario de Jerez de La Frontera, Ronda de Circunvalación S/N, 11407 Jerez de La Frontera, Spain; 2 Department of Biochemistry and Molecular Biology, Faculty of Medicine, University of Cádiz, 11003 Cádiz, Spain; 3 Faculty of Medicine, Department of Medicine, University of Cádiz, INiBICA, 11003 Cádiz, Spain; 4 Department of Medicine, Intensive Care Unit at Hospital Universitario de Jerez de La Frontera, University of Cádiz, INiBICA, 11003 Cádiz, Spain

**Keywords:** Clinical Competence, Communication-methods, Decision Making Clinical, Education Medical-methods, Ethics Medical Professionalism, Palliative Care-methods, Terminal Care-education

## Abstract

End-of-life care training is a critical component of medical education, yet gaps remain in adequately preparing students for these complex interactions. Objective Structured Clinical Examinations (OSCEs) have emerged as a vital tool to assess clinical competence in real-world scenarios. This study aimed to evaluate medical students’ competencies in palliative care using a specialized OSCE station focused on end-of-life skills. A cohort of 118 final-year medical students at the University of Cádiz completed an OSCE that tested abilities in diagnosis, communication, ethical decision-making, and professionalism. Students demonstrated high proficiency in diagnostic competencies, particularly in identifying underlying pathologies and managing refractory symptoms. However, significant variability emerged in skills related to communication, managing advance directives, and obtaining informed consent for palliative sedation. Statistical analyses revealed areas of both strength and challenge, with items related to ethical-legal considerations showing higher difficulty and variability among students. Our findings underscore the need for curricular enhancements that integrate technical training with ethical and communicative aspects of palliative care. Improving student preparation in these domains is essential to equip future physicians with the holistic skills necessary for compassionate end-of-life care.

## Background

End-of-life care (EOLC) training is a critical component of medical education, equipping future physicians to navigate the complex, often ethically charged, realities of palliative care. While studies over the past two decades indicate progress in this area, significant gaps remain in adequately preparing medical students to handle these complex situations. For instance, a longitudinal study of US medical students revealed improvements in the perceived adequacy of EOLC education between 1998 and 2006, with increased percentages of students feeling their training on death and dying was adequate. Despite these advances, recent evidence highlights persistent gaps in exposure and readiness among students [1].

Several barriers limit effective EOLC training in medical education. A qualitative study reported that many students have limited, heterogeneous exposure to dying patients, resulting in a lack of consistent clinical experiences necessary for developing competence in end-of-life care. In some cases, less than half of surveyed students had direct experience with dying patients, with many feeling unprepared to manage symptoms common in palliative settings, such as pain, nausea, and anxiety [2]. Additionally, structured debriefing sessions following encounters with dying patients remain rare in many programs, hindering students' reflective learning in this area [3].

Given these challenges, medical education programs have begun implementing innovative approaches to enhance EOLC training. Programs at institutions like Yale School of Medicine now integrate EOLC instruction longitudinally across all years of study, combining interprofessional learning and skill-building activities with experiential simulations. This integrated approach underscores the need for a comprehensive curriculum that emphasizes both technical competence and ethical sensitivity in end-of-life care [4].

Contemporary medical education faces the constant challenge of adapting its teaching and assessment methods to effectively prepare future physicians in the compassionate and technical management of all phases of illness, especially end-of-life care [5]. Within this context, Objective Structured Clinical Examinations (OSCEs) have been established as a gold standard for assessing clinical skills, integrating theoretical knowledge and practical applications in a rigorous and realistic format [6]. OSCE enables educators to measure clinical competence in controlled environments that simulate real medical situations, providing a comprehensive assessment of students' diagnostic, decision-making, and communication skills [7].

In the field of palliative care, medical training must address not only the relief of physical suffering but also the complex interaction of emotional, social, and ethical factors affecting patients and families [8]. The evaluation using clinical simulation scenarios as tools resembles real clinical practice in a more reliable way than other types of evaluations classically used in the faculties of Health Sciences. Since palliative care requires a multidisciplinary and highly empathetic approach, implementing specific OSCEs for this specialty is essential to ensure adequate training [9].

The novelty of the “End-of-Life Situation” station lies in its integrative approach, assessing not only medical knowledge and practical skills but also students' ability to navigate the emotional and ethical complexities inherent in end-of-life care [10]. This station provides a platform for students to demonstrate their ability to make accurate diagnoses, manage refractory symptoms, effectively communicate with patients and families, and make informed ethical decisions, all crucial elements in palliative practice.

This study aims to address these educational gaps by assessing the end-of-life care competencies of medical students through a specialized OSCE station. By evaluating students' skills in areas such as diagnosis, communication, ethical decision-making, and professionalism, this study seeks to provide insights into the effectiveness of current training and identify specific areas for curricular improvement.

## Methods

Data was systematically collected from a total of 118 medical students, required prior to graduation, meeting the criteria for participating in an OSCE designed to evaluate palliative care competencies at the University of Cádiz in May 2023. The OSCE included a series of 25 assessment items focused on various clinical skills and communication abilities essential in palliative care.

One of the ten stations in the OSCE was specifically designed to evaluate end-of-life care competencies. This station was structured to simulate a real-world scenario and included three key participants: two trained actors (one portraying a patient with a terminal illness and the other a concerned family member) and a qualified evaluator who observed the interaction without intervening. The scenario was carefully scripted to ensure standardization, with the actors adhering to predefined roles that emphasized specific challenges related to end-of-life care, such as managing intense emotions, navigating ethical dilemmas, and making informed decisions about palliative sedation.

Students were tasked with engaging in a structured clinical encounter, which included taking a medical history, assessing the patient’s clinical and emotional status, and addressing the concerns of the family member. During the interaction, students were expected to demonstrate diagnostic proficiency, empathetic communication, and professionalism. For example, the patient actor might express physical discomfort or anxiety about their prognosis, while the family member actor could pose questions about advance directives or request clarity on the patient’s care plan.

The evaluator used a checklist of 25 predefined assessment items to rate the student’s performance across domains such as diagnostic accuracy, empathy, professionalism, and ethical decision-making. This standardized approach minimized subjectivity and ensured consistency across all evaluations. To replicate the unpredictability of real clinical encounters, students were not informed of the specific scoring criteria beforehand. This design provided a realistic assessment of their preparedness to handle complex, emotionally charged scenarios in end-of-life care.

Student performance in the end-of-life care OSCE station was analyzed using basic descriptive statistics, including mean, median, interquartile range, minimum, and maximum scores, to capture central tendencies and distribution across the evaluated competencies. A histogram was created to visually represent score distribution, helping to identify common performance trends.

Each item’s compliance rate was calculated to determine the proportion of students who met specific predefined criteria in the OSCE station. This metric provided a direct measure of how successfully students demonstrated the required competencies, such as diagnosing core pathologies or navigating ethical decision-making. High compliance rates indicated areas where students generally performed well, reflecting the effectiveness of current training in those domains. Conversely, low compliance rates highlighted specific competencies where additional curricular emphasis may be needed.

The discriminative ability of each item was assessed using discrimination coefficients, which measure how effectively an item distinguishes between students with higher and lower overall performance. Items with high discrimination coefficients, such as those assessing communication and consent for palliative sedation, were critical for identifying nuanced competencies and advanced decision-making skills. Items with low discrimination coefficients, where nearly all students answered correctly, typically reflected foundational knowledge or skills that were uniformly mastered and thus required less focused discussion.

Item difficulty was evaluated using ease rates, defined as the proportion of students who successfully completed each item. This measure identified tasks that were inherently more challenging, such as managing advance directives or identifying signs of imminent end-of-life conditions. Together, these metrics—compliance rate, discrimination coefficients, and ease rates—provided a robust framework for interpreting student performance, enabling targeted improvements in training that address specific gaps while reinforcing existing strengths.

All data analyses were performed using SPSS 22.0 for Windows (SPSS Inc., Chicago, IL, USA).

In our evaluation, items related to medical history, examination, communication skills, ethical-legal aspects, and professionalism were analyzed (Table 1).

**Table 1:** Assessment of medical competencies. Total score for all items: 100 points

Item	Value	Description
P1	4	Identifies underlying pathology
P2	6	Identifies refractory symptom
P3	6	Rules out reversible causes
P4	4	Functional, cognitive, socio-familial situation
P5	6	Assesses level and content of consciousness
P6	4	Assesses vital signs
P7	6	Identifies signs on examination suggestive of end-of-life situation (lividity, acral cyanosis, pinched nose, delirium…)
P8	4	Performs cardiac and respiratory auscultation
P9	1.5	Empathy: In the face of intense emotions from the patient (pain, anxiety, joy), participates and empathizes or understands them to redirect them
P10	1.5	Appearance: Well-groomed appearance, good hygiene, correct body posture
P11	1.5	Listening: Appropriate listening, does not interrupt, is attentive, maintains eye contact while speaking
P12	1.5	Cordiality: Provides a warm welcome, smiles
P13	1.5	Respect: Never criticizes or makes pejorative judgments
P14	1.5	Calmness: Remains calm, emotionally composed
P15	1.5	Optimism: Sees the positive aspects of situations, attempts to encourage the patient
P16	1.5	Contact: Physical contact in physical examination or greeting is careful and friendly
P17	1.5	Interest: Shows interest in opinions, beliefs, values, concerns, and emotions
P18	1.5	Expression: Expresses oneself in a way that is clearly understood throughout
P19	8	Identifies refractory symptom despite optimized medical treatment
P20	6.5	Identifies advanced and irreversible underlying pathology
P21	6	Identifies if there is documentation of advance directives
P22	6	Identifies if the patient is capable of making decisions
P23	6.5	Makes the decision to initiate palliative sedation
P24	6	If deciding on palliative sedation, obtains consent from the patient (still capable and conscious)
P25	6	Describes drugs to be used (midazolam and morphine)

## Results

**Fig. 1:**
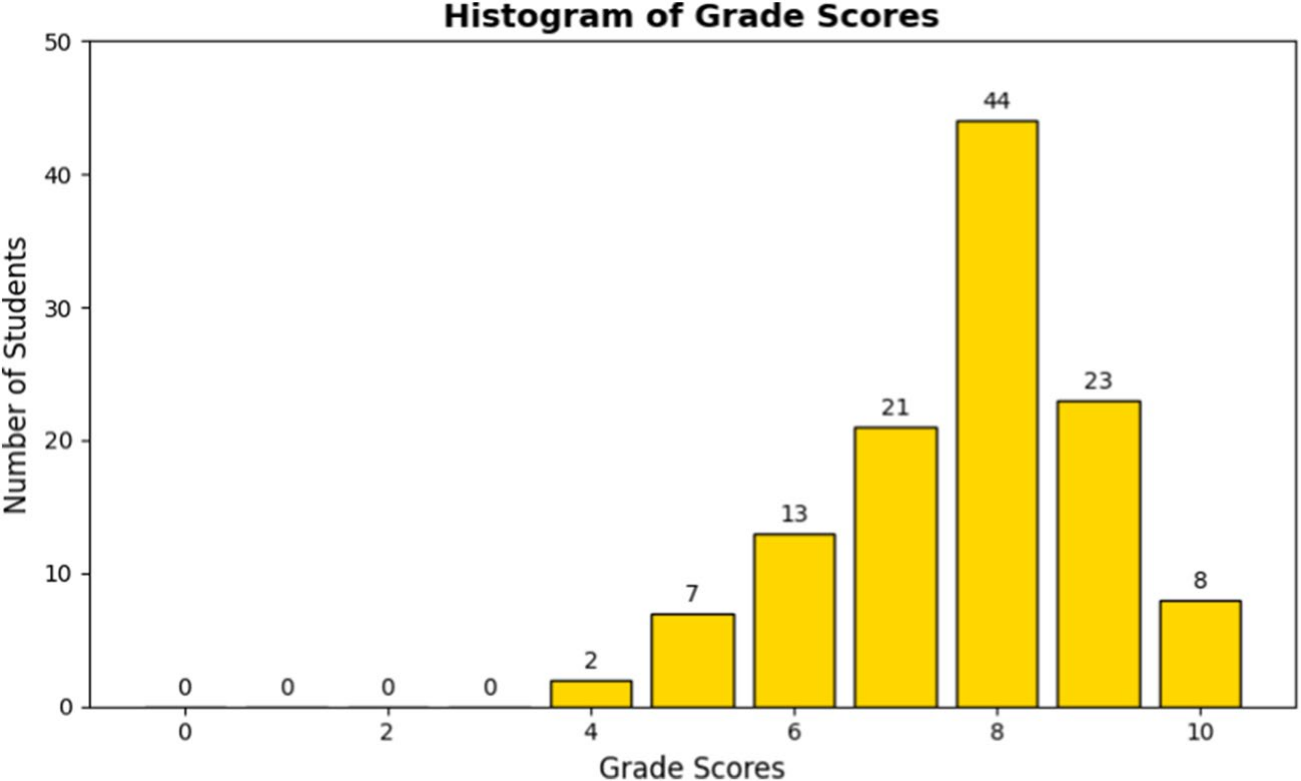
Histogram reflecting the grade scores (*X* axis) obtained by the students (*Y* axis) as whole numbers (from 0 to 10)

A total of 118 students were examined. The mean grade was 8.13, with a median of 8.4 (interquartile range of 7.41–9). The lowest recorded grade was 4.7, while the highest was a 10. The collected data is visualized in the histogram (Fig. 1), which reveals a distribution with a significant mode at the 8 grade mark. However, it is noteworthy that scores are distributed across the entire range of evaluation.

### Compliance with Each Item

**Fig. 2:**
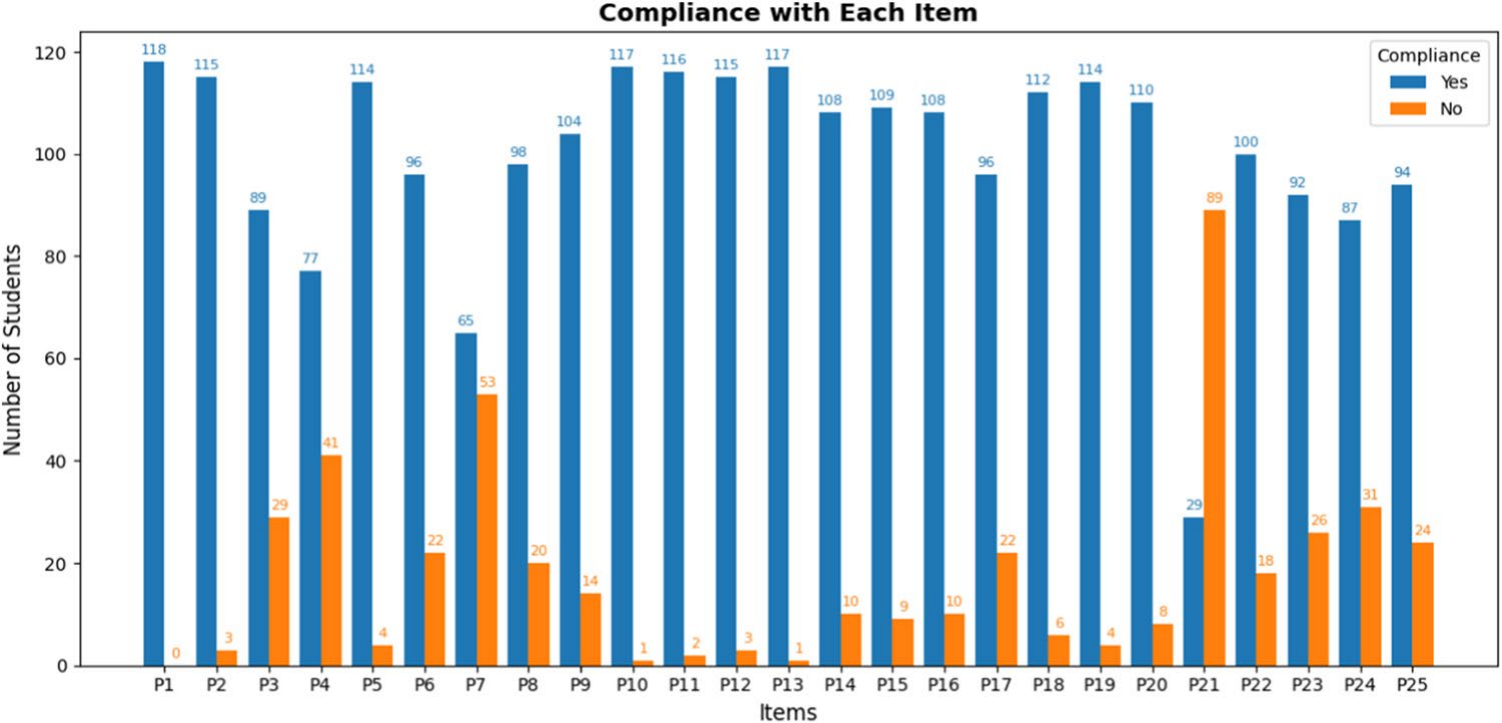
Representation of compliance (blue) or non-compliance (orange) for each of the items

The results obtained provide a detailed quantitative perspective on the degree of compliance with the established learning objectives (Fig. 2).

The analysis of compliance with individual OSCE items revealed both high and low-performing areas among the students. Specifically:

- **Highest compliance**: Item P1, “Identification of underlying pathology,” achieved a 100% compliance rate, indicating that all students successfully met this criterion. This suggests strong foundational knowledge in diagnosing core pathologies, a critical component in end-of-life care.
- **High compliance in core skills**: Other items with high compliance rates include item P5 (“Assessment of level and content of consciousness”) with 96.6% compliance and item P8 (“Performance of cardiac and respiratory auscultation”) with 88.1%. These results underscore students’ proficiency in core clinical assessments.
- **Moderate compliance**: Items such as P3, “Ruling out reversible causes,” and P4, “Assessment of functional, cognitive, and socio-familial situation,” showed lower yet moderate compliance rates of 75.4% and 65.3%, respectively. These results highlight areas where students demonstrated adequate but less consistent performance.
- **Lowest compliance**: Item P21, “Identification of advance directives documentation,” had the lowest compliance rate at 24.6%. This result indicates a significant gap in students’ ability to recognize and manage end-of-life planning documents.

### Discriminative Ability of the Items

**Fig. 3:**
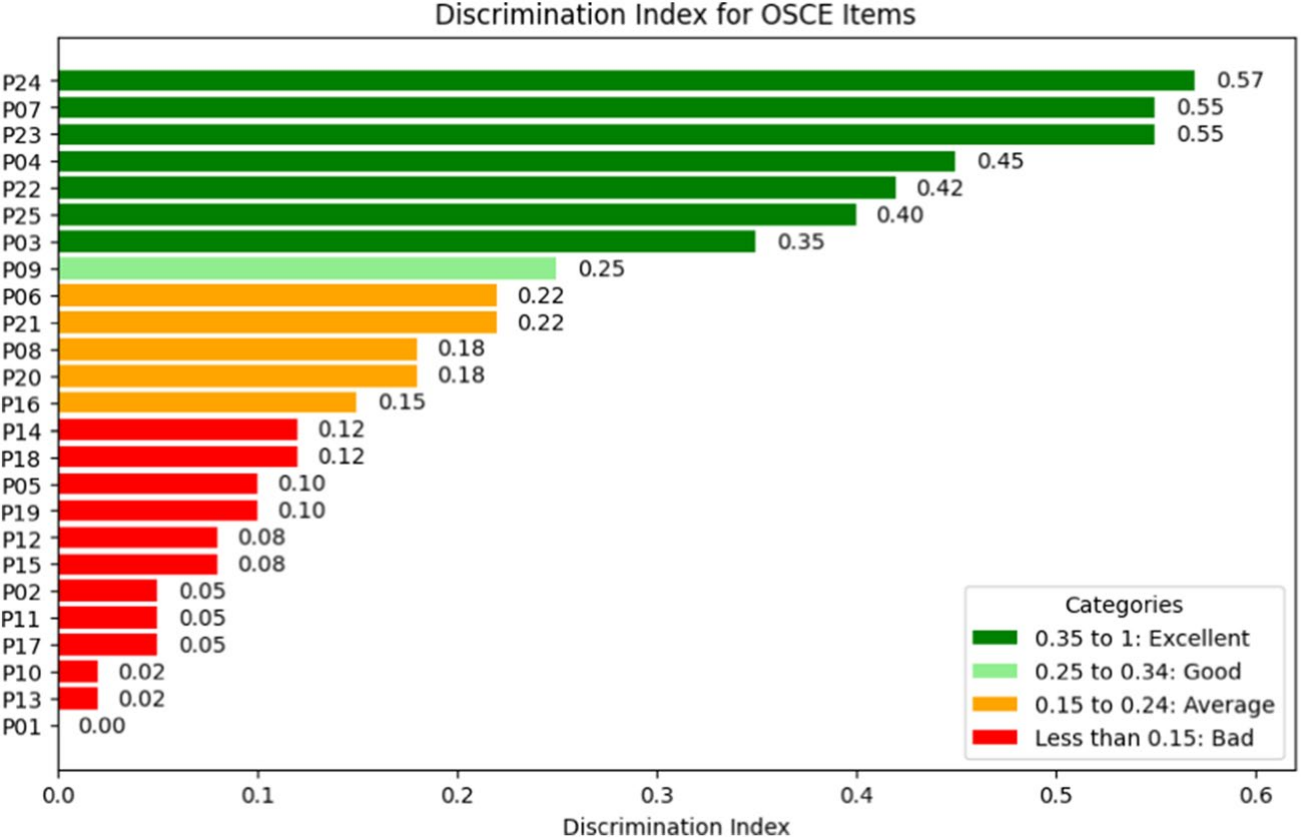
Representation of the discriminative capacity of each of the items

In our OSCE station analysis, this capacity was measured for each evaluated item to identify those most indicative of superior understanding and skill among the students (Fig. 3).

Item P24, “obtaining patient consent for palliative sedation,” demonstrated the highest discriminative ability with a coefficient of 0.57. Following P24, items P7 (“identification of signs on examination suggestive of end-of-life situation”) and P23 (“decision to initiate palliative sedation”) showed strong discrimination coefficients of 0.55 and 0.45, respectively, reinforcing the need for curriculum components that address these nuanced clinical judgment areas.

Moderate discrimination values were observed in items like P3, “ruling out reversible causes,” and P4, “assessment of functional, cognitive, and socio-familial situation,” with coefficients of 0.42 and 0.4, respectively. Conversely, some items exhibited low discrimination, which is expected in widely correct items. Items with the lowest discrimination, such as P17 (“interest in patient opinions and emotions”) and P13 (“respect”), had coefficients as low as 0.05.

### Difficulty

**Fig. 4:**
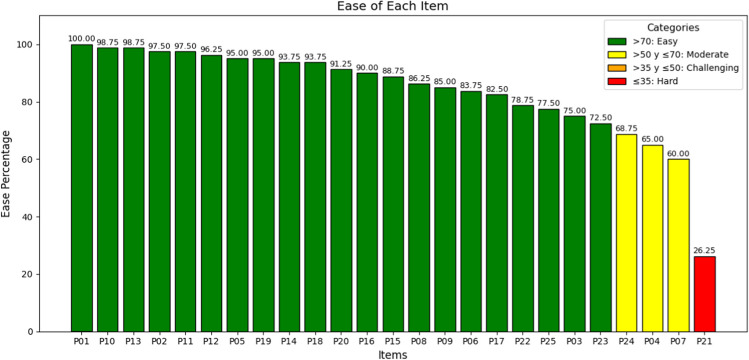
Representation of the ease of each of the items

The evaluation of item difficulty constitutes a crucial aspect for understanding the areas that pose greater challenges for students in the ECOE station. This analysis allows us to discern which components of the evaluation require a more advanced mastery or intensive preparation (Fig. 4).

Among the evaluated items, item P21, “identifies if there is documentation of advance directives,” emerged as the most challenging, with an ease rate of 26.25%. Other items that proved complex for the students included P7, “identification of signs on examination suggestive of end-of-life situation,” and P24, “obtaining patient consent for palliative sedation,” with ease rates of 60% and 65%, respectively.

In contrast, item P1, “identification of underlying pathology,” showed an ease rate of 100%, implying that all students were able to fulfill this requirement without difficulty. Items related to communication and behavioral skills, such as P9, “empathy towards intense emotions of the patient,” P10, “well-groomed appearance and correct body posture,” and P11, “appropriate listening,” also presented high ease rates.

It is important to note that the distribution of difficulty was not homogeneous across all items, underscoring the heterogeneity in training and mastery of specific skills. For example, P3, “ruling out reversible causes,” and P4, “assessment of functional, cognitive, and socio-familial situation,” showed ease rates of 78.75% and 68.75%, respectively.

## Discussion

The detailed assessment of results by items in the OSCE station reveals significant trends in the clinical and communicative competence of medical students. This discussion explores potential explanations for these findings, offering an interpretative framework grounded in the evidence provided.

Adequate preparation in palliative care requires a multidisciplinary approach that integrates medical knowledge, communication skills, and ethical competencies [11]. The results obtained in the OSCE station may reflect factors inherent to the complex nature of end-of-life care. Some items, such as P1, showed high overall compliance but low discrimination scores. This pattern is typical for items that nearly all students answer correctly, as there are fewer differences among students' responses. Low discrimination scores in these cases confirm that foundational competencies were widely achieved, which means further detailed discussion of these items is unnecessary.

The difficulty of other items highlights specific gaps in training. Item P21, identified as the most challenging, pertains to understanding and managing advance directives, a critical aspect of advance care planning in palliative medicine. This challenge reflects a medical reality in which students may encounter barriers to openly discussing end-of-life wishes with patients [12]. Despite their clinical and ethical importance, advance directives are often underemphasized in medical training [13]. The difficulty observed in this item suggests a gap between theoretical knowledge and practical application, underscoring the need for more focused training in advanced communication skills and shared decision-making. Strengthening curriculum components related to end-of-life documentation and patient-centered discussions could help bridge this gap, aligning with the principles of modern palliative medicine [14].

In contrast, the high discrimination score of item P24 (“obtaining informed consent”) highlights the complexity and importance of navigating decisions with high emotional and ethical stakes. In the palliative context, obtaining informed consent involves aligning treatment choices with patient values, which requires sensitivity, legal understanding, and reflective practice [15]. Students who excelled in this item likely demonstrated strong communication skills and empathy, critical skills for palliative care. This finding suggests that curricular adjustments should include explicit instruction on end-of-life discussions and informed consent, potentially incorporating simulations, small-group discussions, and case-based reflections [16]. Emphasizing not only what to communicate but also how to do so in a way that respects patient dignity and preferences is central to quality palliative care [17].

The high discrimination value of P24 underscores its utility in distinguishing students who are ready for more complex clinical interactions [18] and highlights the importance of integrating ethical and communicative skills with clinical knowledge in palliative education [19, 20].

These results suggest several targeted improvements to the curriculum. A more structured emphasis on advance care planning and documentation could better prepare students for real-world interactions on these critical topics [21]. Additionally, training that focuses on obtaining informed consent in high-stakes situations, such as decisions regarding palliative sedation, would enhance students’ capacity to handle these sensitive discussions [22]. Scenarios involving family discussions about end-of-life care can help students develop empathy and professionalism, essential qualities in palliative care [23].

Implementing similar OSCE stations at other institutions would provide valuable, hands-on training in end-of-life care. However, this approach requires resources, including trained evaluators, standardized patients, and realistic clinical scenarios. Proper examiner training and standardized assessment protocols are essential to ensure consistency and objectivity across institutions. Despite the logistical demands, OSCE stations focused on palliative care provide experiential learning that could be applied across diverse medical training programs [18].

The variability in compliance with communication and behavioral skills (e.g., items P9–P18) underscores the need for a patient-centered approach in medical education. Skills such as empathy, respect, and effective communication are not merely complementary to technical training; they are essential for high-quality palliative care. Performance in these items suggests that additional training in interpersonal skills could benefit students, possibly through a more reflective, patient-centered pedagogy [24]. A teaching approach that fosters reflection and interpersonal development, combined with assessment methods that accurately reflect clinical competence, would support students in acquiring the holistic skills essential for effective palliative care [25].

In summary, the OSCE results illustrate the importance of a comprehensive approach to palliative care education, balancing clinical rigor with the development of empathetic and ethical competencies. By incorporating these curriculum adjustments, medical programs can better prepare future physicians to provide compassionate and competent end-of-life care, honoring patients’ dignity and preferences at all stages of illness [26, 27].

## Conclusions

The assessment conducted through the “End-of-Life Care” OSCE has provided valuable insights into the competencies of medical students in palliative care, highlighting both their strengths and the areas needing further development. The results demonstrate a strong proficiency in essential diagnostic skills, affirming students’ foundational knowledge. However, significant challenges were identified in critical areas such as the management of advance directives and the ability to obtain informed consent for complex procedures like palliative sedation.

The variability observed in communication skills and ethical decision-making, particularly concerning patient consent, underscores the need for curricular enhancements that focus on these components. This aligns with existing literature on training gaps in end-of-life care, where a balance between technical skill development and ethical, patient-centered communication is crucial. Addressing these gaps will require curricula that explicitly integrate ethical principles, communication strategies, and shared decision-making exercises, preparing students to manage complex and sensitive interactions with confidence and empathy.

In conclusion, this study highlights the ongoing need for educational programs that equip future physicians with not only the technical but also the ethical and interpersonal skills necessary for high-quality palliative care. A holistic and compassionate approach in medical education is essential to produce professionals who can provide comprehensive, sensitive, and dignified care, respecting the needs and preferences of patients at all stages of illness.

## Data Availability

Data supporting reported results can be shared under request at cristilc2@gmail.com
